# Online Advertising of Compounded Glucagon-Like Peptide-1 Receptor Agonists

**DOI:** 10.1001/jamahealthforum.2024.5018

**Published:** 2025-01-17

**Authors:** Ashwin K. Chetty, Mahima Chillakanti, Reshma Ramachandran, Joseph S. Ross, Alissa S. Chen

**Affiliations:** 1 Yale University School of Medicine, New Haven, Connecticut; 2 David Geffen School of Medicine at the University of California Los Angeles, Los Angeles; 3 Section of General Internal Medicine, Department of Internal Medicine, Yale University School of Medicine, New Haven, Connecticut; 4 Yale Collaboration for Regulatory Rigor, Integrity and Transparency, Yale University School of Medicine, New Haven, Connecticut; 5 Department of Health Policy and Management, Yale University School of Public Health, New Haven, Connecticut; 6 Center for Outcomes Research and Evaluation, Yale-New Haven Health System, New Haven, Connecticut; 7 National Clinician Scholars Program, Department of Internal Medicine, Yale University School of Medicine, New Haven, Connecticut

## Abstract

This cross-sectional study evaluates the quality and accuracy of information presented on websites that sell compounded glucagon-like peptide-1 receptor agonists.

## Introduction

High global demand for glucagon-like peptide-1 receptor agonists (GLP-1 RAs) and dual gastric inhibitory polypeptide/GLP-1 RAs has exceeded manufacturers’ supply, leading to medication shortages that have persisted since 2022.^1^ Medication shortages allow pharmacies to sell compounded versions of US Food and Drug Administration (FDA)–approved drugs, including GLP-1 RAs. Compounded medications contain the same active ingredients as in branded medications but may contain different inactive ingredients and are not FDA approved.^2,3^ US federal law requires advertising of all prescriptions, including compounded medications, to be “truthful, non-misleading, and accurate”^2^; however, the extent to which compounded medication advertising meets this mandate is unclear. Nonmisleading advertising is important given that 11% of GLP-1 RA recipients obtain their GLP-1 RAs through a website^4^ and given past examples of misleading advertising.^3^ Therefore, we assessed advertising practices of websites selling compounded GLP-1 RAs, including semaglutide, tirzepatide, and liraglutide.

## Methods

Two of us (A.K.C., M.C.) conducted online searches between July and September 2024 to identify websites advertising the sale of compounded semaglutide, tirzepatide, or liraglutide or prescriptions for these GLP-1 RAs. Searches using brand and generic medication names were conducted using Google Shopping (Alphabet Inc); all websites appearing in Google’s sponsored results were included (eMethods in Supplement 1). In accordance with the Common Rule (45 CFR §46), this cross-sectional study was exempt from ethics review and informed consent because it was not human participant research. We followed the STROBE reporting guideline.

Websites were reviewed and data were extracted using a structured form (eTable in Supplement 1); disagreements between reviewers were resolved by consensus. Extracted information included location, medications sold and associated prices, compounding disclosures, efficacy and safety claims, use of brand names, and clinician involvement. Descriptive statistics were calculated using R 4.2.3 (R Project for Statistical Computing).

## Results

During the study period, 98 unique websites sold any GLP-1 RA, of which 79 sold compounded GLP-1 RAs or a prescription for compounded medications and were included in the analysis. All 79 websites sold compounded semaglutide, 57 (72.2%) sold compounded tirzepatide, and 3 (3.8%) sold compounded liraglutide (Table 1). Median (IQR) first-month price, including discounts, for compounded semaglutide, tirzepatide, and liraglutide was $231 ($189-$294), $330 ($275-$399), and $248 ($223-$274), respectively. Fifty-two websites (65.8%) featured a mark of certification, 50 of which displayed LegitScript certification. Two websites (2.5%) did not provide prescriptions for compounded GLP-1 RAs and required a prior prescription.

**Table 1.** Website and Medication Information on Websites Selling Compounded Liraglutide, Semaglutide, or Tirzepatide

Website or medication characteristic	Websites, No. (%) (N = 79)
Accreditation, certification, or equivalent claim^a^	52 (65.8)
Location of sales	
Online only	56 (70.9)
In person only	6 (7.6)
Online and in person	17 (21.5)
Medications sold	
Compounded liraglutide	3 (3.8)
Compounded semaglutide	79 (100)
Compounded tirzepatide	57 (72.2)
Branded GLP-1 RA	23 (29.1)
Compounded GLP-1 RA with a supplement	29 (36.7)
Does not sell salt form^b^	16 (20.3)
Routes of administration	
Injectable	78 (98.7)
Sublingual	10 (12.7)
Oral	1 (1.3)
Clinician involvement required to obtain compounded GLP-1 RA^c^	
Any clinician involvement^d^	78 (98.7)
Completion of a questionnaire reviewed by a clinician	50 (63.3)
Messaging with a clinician	3 (3.8)
Telehealth visit (telephone call or video)	31 (39.2)
Virtual contact but method is unspecified	20 (25.3)
In-person visit	5 (6.3)
Prior prescription; website does not provide prescriptions	2 (2.5)
Labwork	8 (10.1)

Abbreviation: GLP-1 RA, glucagon-like peptide-1 receptor agonist.

^a^ Accreditation, certification, or equivalent claim was featured on the website via text, image, or video. Most websites reported certification from LegitScript, an organization that states it “provides a recognized stamp of approval for businesses that facilitate transactions for pharmacies.” It helps businesses adhere to pharmacy and telemedicine regulations, and its certification indicates to patients that a business is operating “safely and legally.”

^b^ Salt form (eg, semaglutide acetate or semaglutide sodium) includes a different active ingredient than used in the US Food and Drug Administration–approved branded GLP-1 RA.

^c^ Some websites offered multiple pathways to receive a prescription, such as a telehealth or an in-person visit, or required telehealth only if mandated by state law. In these cases, the least involved pathway to obtaining a prescription was recorded.

^d^ For 1 website, it was unclear whether any clinician involvement was required, and there was no mention of certain requirements to obtain a compounded GLP-1 RA.

Eleven websites (13.9%) did not disclose the GLP-1 RAs for sale were compounded, while 7 (8.9%) referred to compounded medications as generic (Table 2). While 34 websites (43.0%) stated the compounded medications were not FDA approved, 29 (36.7%) stated or implied these drugs were FDA approved. Thirty-nine websites (49.4%) did not report adverse effects, warnings and precautions, and contraindications of compounded GLP-1 RAs, whereas 32 (40.5%) advertised an efficacy claim not in the authorized label of the FDA-approved branded GLP-1 RA.

**Table 2.** Advertising Information on Websites Selling Compounded Liraglutide, Semaglutide, or Tirzepatide

Advertising characteristic	Websites, No. (%) (N = 79)
Compounding disclosures	
Mentions at least once that medication is compounded	68 (86.1)
Consistently mentions that medication is compounded^a^	13 (16.5)
Provides definition of compounding^b^	39 (49.4)
Refers to compounded medication as generic	7 (8.9)
States compounded medication is not FDA approved	34 (43.0)
States or implies compounded medication is FDA approved	29 (36.7)
States there are FDA-approved GLP-1 RAs	45 (57.0)
Claims accreditation, certification, or equivalent for partner compounding pharmacy^c^	58 (73.4)
Safety information	
Clinical trial evidence to support safety claims	13 (16.5)
Link to FDA label^d^	18 (22.8)
Adverse effects^e^	68 (86.1)
Contraindications^e^	50 (63.3)
Warnings and precautions^e^	49 (62.0)
Efficacy information	
Clinical trial evidence to support efficacy claims	65 (82.3)
Efficacy claim not included in labels of FDA-approved branded GLP-1 RAs	32 (40.5)
Effects of discontinuation	29 (36.7)
Use for weight management	79 (100)
Use for glucose management	69 (87.3)
Use for cardiovascular risk reduction	36 (45.6)
Use of brand name	
In advertising	62 (78.5)
Use of “same active ingredients as [brand name]” or equivalent phrase	50 (63.3)
Use of image of brand-name medication to represent compounded medication	1 (1.3)

Abbreviations: FDA, Food and Drug Administration; GLP-1 RA, glucagon-like peptide-1 receptor agonist.

^a^ *Consistently* was defined as always referring to the compounded GLP-1 RA as compounded within the same sentence or phrase.

^b^ *Compounding* was defined either with a link to an FDA web page about compounded GLP-1 RAs or any statement using the terms *compounding, compounded*, or a variation and inclusion of some part of this definition from the Federal Food, Drug, and Cosmetic Act: “…combining, admixing, mixing, diluting, pooling, reconstituting, or otherwise altering of a drug or bulk drug substance to create a drug.”

^c^ A subset of websites disclosed the specific type of compounding pharmacy they worked with and/or specific information about these facilities. Ten websites worked with 503B pharmacies, and 9 websites worked with 503A pharmacies.

^d^ If a website linked to at least 1 FDA label for any branded liraglutide, semaglutide, or tirzepatide, then this field was marked yes.

^e^ If a website mentioned any adverse effects, contraindications, or warnings and precautions, then this field was marked yes.

## Discussion

This cross-sectional study showed websites that sell compounded GLP-1 RAs often partially informed and sometimes misinformed potential consumers. Most websites did not disclose that compounded GLP-1 RAs were not FDA approved, although some suggested these drugs were FDA approved. Many websites provided limited safety information and unauthorized efficacy claims. Some websites did not disclose that these medications were compounded or incorrectly referred to them as generic.

A study limitation is that search results varied by location. Moreover, because we reviewed all information reported on websites, including on subpages and associated blogs, we likely overestimated the amount of information that typical consumers review.

Enhanced regulatory guidance and oversight are needed to clarify criteria for “truthful, non-misleading, and accurate”^2^ advertising to ensure consumers are informed of the risks and benefits of compounded GLP-1 RAs and other compounded medications. The FDA could require websites to explicitly disclose and define compounding, including lack of FDA approval; institute unique naming conventions for compounded medications; and be given greater authority to act against misleading compounded medication advertising.^5,6^

## Notes

Supplement 1. **eMethods.** Methodological Details **eTable.** Structured Form
eReferences

Supplement 2.
Data Sharing Statement
