# Supplementary material for: Online Advertising of Compounded Glucagon-Like Peptide-1 Receptor Agonists
Source: JAMA Health Forum. 2025 Jan 17;6(1):e245018. doi: 10.1001/jamahealthforum.2024.5018 (PMC11742527; doi:10.1001/jamahealthforum.2024.5018)
Supplement: Supplement 1. — eMethods. Methodological Details eTable. Structured Form eReferences [file jamahealthforum-e245018-s001.pdf]

Supplemental Online Content

Chetty AK, Chillakanti M, Ramachandran R, Ross JS, Chen AS. Online advertising of compounded glucagon-like peptide-1 receptor agonists. *JAMA Health Forum.* Published online January 17, 2025. doi:10.1001/jamahealthforum.2024.5018

eMethods. Methodological Details

eTable. Structured Form

eReferences

This supplemental material has been provided by the authors to give readers additional information about their work.

© 2025 Chetty AK et al. *JAMA Health Forum*.

eMethods. Methodological Details

Google Search. Two independent reviewers (A.K.C. and M.C.) using the Google Chrome web browser turned off personalized ads (My Ad Center → Personalized ads = Off) and turned off personalized results (settings → Search settings → Personal results = Off). The reviewers executed the following search strategies in the Google Search engine Shopping and All tabs:

Google Search Shopping tab

"semaglutide OR tirzepatide OR liraglutide OR wegovy OR zepbound OR saxenda OR ozempic OR rybelsus OR mounjaro OR victoza"

Google Search All tab

"(semaglutide OR tirzepatide OR liraglutide OR wegovy OR zepbound OR saxenda OR ozempic OR rybelsus OR mounjaro OR victoza) AND inurl:.com -site:youtube.com"

The phrase "inurl:.com -site:youtube.com" limits search results to URLs ending in ".com" and excludes video results from YouTube. Each reviewer, one located in Los Angeles, CA and another located in New Haven, CT, executed both searches.

Structured form. A structured form on Microsoft Forms (eTable) was completed for all websites meeting study inclusion criteria: English-language websites selling compounded semaglutide, liraglutide, or tirzepatide, or selling a prescription for these compounded drugs. Only the first section of the structured form pertaining to website information was completed for websites selling non-compounded versions of semaglutide, liraglutide, or tirzepatide. Generic liraglutide was included among non-compounded versions.

Some websites sold a compounded GLP-1RA but did not disclose anywhere that it was compounded. For these websites, we assessed if they advertised a compounded GLP-1RA based on using verbiage suggesting that the GLP-1RA was not a branded GLP-1RA or not generic liraglutide (e.g. use of euphemisms for compounding, no mention of branded names, price discordant with branded GLP-1RAs or generic liraglutide, lack of website certification).

All answers to the structured form were based on text, image, and video media available on each website, including subpages and associated blogs. Reviewers did not register accounts on websites, contact websites or associated providers, or try to purchase the compounded GLP-1RAs. Consumers who purchase the compounded GLP-1RAs may or may not receive additional information during the purchasing process.

© 2025 Chetty AK et al. *JAMA Health Forum.*

Variables.

Variables	Definition
States the website/seller is accredited, certified, or an equivalent claim	Website features a statement in text or video, or an image/sticker, indicating accreditation or certification of the website from another organization.
**Location of sales**	
Sales of compounded GLP-1RA online only	Location of sale was defined by where a potential consumer can receive care and/or the compounded medication. For example, some websites were associated with brick-and-mortar locations, but the website only mentioned remote care and shipping of the compounded medications – this was considered an online location of sale. Some websites required all potential consumers to come in-person for an evaluation before obtaining a prescription – this was considered an in-person location of sale. Some websites offered in-person care or remote care – this was considered both online and in-person.
Sales of compounded GLP-1RA in-person only	
Sales of compounded GLP-1RA online and in-person	
**Medications sold**	
Sells compounded liraglutide	Website sells compounded liraglutide, semaglutide, or tirzepatide or sells a prescription for these GLP-1RAs.
Sells compounded semaglutide	
Sells compounded tirzepatide	
Sells a branded GLP-1RA	Website sells a branded version of liraglutide, semaglutide, or tirzepatide.
Compounds GLP-1RA with a supplement (ex. vitamin B12)	Website offers to add a supplement (e.g. vitamin B12, carnitine, citrulline) to the same formulation as the compounded medication. Some website sold supplements in addition to the compounded medication and encouraged consumers to buy both separately – this was not considered compounding GLP-1RA with a supplement.
States it does not sell a salt form[a] of semaglutide, tirzepatide, or liraglutide	Website explicitly states that it does not sell a salt form of a compounded GLP-1RA, or explicitly states that it sells a base form of a compounded GLP-1 RA.
First-month price	First-month price was calculated with the inclusion of initiation discounts. The cost of any required program membership was excluded if the website stated this cost – some websites may have combined the cost of medication and membership without

© 2025 Chetty AK et al. *JAMA Health Forum.*

	disclosing membership cost. Some websites required purchasing more than one month of supply at initiation, in which case the first-month price was prorated. Since reviewers did not attempt to purchase the drugs, potential consumers may face additional fees during the purchasing process that websites do not initially disclose.
**Routes of administration**	
Sells injectable GLP-1RA	The route of administration offered was based on information provided in text, images, and videos. Some websites marketed a compounded GLP-1RA as oral but clarified on subpages that it was a sublingual route of administration – this was recorded as a sublingual offering.
Sells sublingual GLP-1RA	
Sells oral GLP-1RA	
**Compounding Disclosures**	
States medication is compounded, at least once	Website uses the word "compounded" or a variation to describe its version of liraglutide, semaglutide, or tirzepatide at least once.
Consistently refers to medication as compounded	Website always refers to the compounded GLP-1RA as compounded within the same sentence or phrase.
Provides definition of compounding	A definition of compounding was considered a link to an FDA webpage about compounded GLP-1RAs, or any statement that used the term "compounding," "compounded," or a related variation and included some form of the following definition from the Federal Food, Drug, and Cosmetic Act: "The term "compounding" includes the combining, admixing, mixing, diluting, pooling, reconstituting, or otherwise altering of a drug or bulk drug substance to create a drug."[1]
Refers to compounded medications as "generic"	Website uses the term "generic" to describe a compounded GLP-1RA, at least once.
States compounded medication is not FDA approved	Website explicitly states that the compounded GLP-1RA, or compounded medications in general, are not FDA approved.
States or implies compounded medication is FDA approved	Website makes statements such as "semaglutide is FDA approved" without any

	clarification that compounded GLP-1RAs are not FDA approved.
States there are FDA-approved GLP-1RAs	Website provides information indicating that there are GLP-1RAs that are FDA approved. Only providing the names of branded GLP-1RAs was not sufficient; the website had to also indicate they are FDA approved.
States compounding pharmacy is accredited, certified, or an equivalent claim	Website indicates that the compounding pharmacy is accredited or certified by another organization.
**Safety Information**	
Cites clinical trial evidence to support safety claims	Website cites at least one published, peer-reviewed clinical trial of GLP-1RAs to provide safety information (e.g. rate of gastrointestinal side effects).
Links to FDA label	Website links to at least one FDA label for any branded version of liraglutide, semaglutide, or tirzepatide.
States adverse effects	Website mentions at least one adverse effect present in an authorized label for an FDA-approved branded GLP-1RA. The same criterion was used for contraindications and for warnings and precautions. Warnings and precautions included mentioning that semaglutide, liraglutide, or tirzepatide causes thyroid C-cell tumors in rodents.
States contraindications	
States warnings and precautions	
**Efficacy Information**	
Cites clinical trial evidence to support efficacy claims	Website cites at least one published, peer-reviewed clinical trial of GLP-1RAs to make an efficacy claim.
Contains an efficacy claim not present in FDA labels for branded GLP-1RAs	Website mentions an efficacy claim that is not present in an authorized label for an FDA-approved branded GLP-1RA.
States effects of GLP-1RA discontinuation	Website provides any information about the potential effects of GLP-1RA discontinuation (e.g. weight regain).
Advertises use for weight management	Website provides information indicating the compounded GLP-1RA can be used for weight management, glucose management, or cardiovascular risk reduction. Uses not on an FDA label for a branded GLP-1RA were not included here. For example, advertising use of tirzepatide for cardiovascular risk reduction was not
Advertises use for glucose management	
Advertises use for cardiovascular risk reduction	

© 2025 Chetty AK et al. *JAMA Health Forum*.

	included here; this was included under "efficacy claim not present in FDA labels."
**Use of Brand Name**	
References brand name medication in advertising	Website mentions the brand name of a GLP-1RA, at least once.
States "same active ingredients as [brand name]" or equivalent phrase	Website states that the compounded GLP-1RA has the same active ingredient as a branded GLP-1RA.
Uses image of brand name medication when advertising compounded medication	Website includes an image of a branded GLP-1RA on a webpage that is selling a compounded GLP-1RA and not selling a branded GLP-1RA.
**Clinician Involvement[b]**	
Requires any clinician involvement to obtain compounded GLP-1RA	Information on website indicates that website requires any level of interaction with a clinician, synchronously or asynchronously, to obtain a GLP-1RA
Requires completing a questionnaire reviewed by clinician	Information on website indicates that website requires completion of a questionnaire to obtain a compounded GLP-1RA.
Requires messaging with clinician	Information on website indicates that website requires messaging with a clinician, synchronously or asynchronously, to obtain a compounded GLP-1RA.
Requires telehealth visit (call or video)	Information on website indicates that website requires a telephone call or video meeting with a clinician to obtain a compounded GLP-1RA.
Requires virtual contact but method is unspecified	Information on website indicates that website requires contact with a clinician but doesn't specify method of contact and does not indicate an in-person visit is required to obtain a compounded GLP-1RA.
Requires in-person visit	Information on website indicates that an in-person visit with a clinician is required to obtain a compounded GLP-1RA.
Requires prior prescription, the website does not provide prescriptions itself	Information on website indicates that the website does not provide a prescription for compounded GLP-1RAs and that prospective consumers must obtain a prescription before purchasing a compounded GLP-1RA through the website.

© 2025 Chetty AK et al. *JAMA Health Forum*.

Requires labwork	Information on website indicates that prospective consumers must obtain labwork (ex. HbA1c, lipid levels, etc.) to obtain a compounded GLP-1RA.

[a] Salt form refers to, for example, semaglutide acetate or semaglutide sodium, which is a different active ingredient than is used in the Food and Drug Administration-approved branded GLP-1RAs. The FDA has warned the public about the use of salt forms of compounded GLP-1RAs and has indicated that compounding with salt forms does not adhere to federal law.[2]

[b] Some websites offered multiple pathways to receive a prescription, such as a telehealth visit or an in-person visit, or only requiring telehealth if a state law required it. In these cases, the least involved pathway to obtaining a prescription was recorded, which could still include multiple requirements such as completing a questionnaire and messaging with a clinician.

GLP-1RA: glucagon-like peptide-1 receptor agonist, or gastric inhibitory peptide/glucagon-like peptide-1 receptor agonist; FDA: Food & Drug Administration

© 2025 Chetty AK et al. *JAMA Health Forum*.

eTable. Structured Form

Question	Answer Format or Choices
Date website was accessed	Open calendar answer
Website URL	Open answer
Website name	Open answer
Is the data on this form extracted by the first or second rater?	1, 2
Which search method was used to find this website?	Google: Shopping Tab; Google: All Tab; Found by other rater
Does the website indicate that it only sells a branded GLP-1RA (only using brand name and image, price concordant with brand name drug, indication of website certification/legitimacy)?	yes, no [if yes, end form]
Does the website sell compounded liraglutide (does NOT include generic liraglutide from Teva Pharmaceuticals)?	yes, no, other
Does the website sell a salt form of liraglutide?	yes, no (explicitly states no), unspecified, NA
What compounded liraglutide routes of administration are offered?	oral, sublingual, injectable, NA, other [multiple selections allowed]
What compounded liraglutide formulations are offered?	tablet, vial, pen, liquid drops, NA, other [multiple selections allowed]
What's the price for the first month of compounded injectable liraglutide? (including initiation discounts) (don't include $ sign or commas)	Open numeric answer
What's the price for the first month of compounded oral liraglutide? (including initiation discounts) (don't include $ sign or commas)	Open numeric answer
What's the price for the first month of compounded sublingual liraglutide? (including initiation discounts) (don't include $ sign or commas)	Open numeric answer
Other information on compounded liraglutide price (discounts, normal price, different prices for sublingual tablet vs. drops, other notable findings, etc.)	Open answer
Does the website sell compounded semaglutide?	yes, no, other
Does the website sell a salt form of semaglutide?	yes, semaglutide acetate; yes, semaglutide sodium; yes, unspecified salt; no (explicitly states no); unspecified, NA

© 2025 Chetty AK et al. *JAMA Health Forum.*

What compounded semaglutide routes of administration are offered?	oral, sublingual, injectable, NA, other [multiple selections allowed]
What compounded semaglutide formulations are offered?	tablet, vial, pen, liquid drops, NA, other [multiple selections allowed]
What's the price for the first month of compounded injectable semaglutide? (including initiation discounts) (don't include $ sign or commas)	Open numeric answer
What's the price for the first month of compounded oral semaglutide? (including initiation discounts) (don't include $ sign or commas)	Open numeric answer
What's the price for the first month of compounded sublingual semaglutide? (including initiation discounts) (don't include $ sign or commas)	Open numeric answer
Other information on compounded semaglutide price (discounts, normal price, different prices for sublingual tablet vs. drops, other notable findings, etc.)	Open answer
Does the website sell compounded tirzepatide?	yes, no, other
Does the website sell a salt form of tirzepatide?	yes, no (explicitly states no), unspecified, NA
What compounded tirzepatide routes of administration are offered?	oral, sublingual, injectable, NA, other [multiple selections allowed]
What compounded tirzepatide formulations are offered?	tablet, vial, pen, liquid drops, NA, other [multiple selections allowed]
What's the price for the first month of compounded injectable tirzepatide? (including initiation discounts) (don't include $ sign or commas)	Open numeric answer
What's the price for the first month of compounded oral tirzepatide? (including initiation discounts) (don't include $ sign or commas)	Open numeric answer
What's the price for the first month of compounded sublingual tirzepatide? (including initiation discounts) (don't include $ sign or commas)	Open numeric answer
Other information on compounded tirzepatide price (discounts, normal price, different prices for sublingual tablet vs. drops, other notable findings, etc.)	Open answer

© 2025 Chetty AK et al. *JAMA Health Forum.*

Does the website offer to add supplements to the compounded medication?	vitamin B12 (cobalamin); other B-complex vitamin(s); chromium, vitamin D; no; other [multiple selections allowed]
Location of sales	online, inperson, both
Does the website sell a branded GLP-1RA in addition to the compounded GLP-1RA?	yes, no
Does the website refer to the proprietary name of the FDA-approved GLP-1RA (in text, image, or video)? (does not apply to selling the branded GLP-1RA)	yes, no
Does the website use the phrase "same active ingredient as [brand name]" or a similar phrase?	yes, no
Does the website use the phrase "compounded [brand name]" or "[brand name]" when referring to the compounded drug?	yes, no
Does the website display an image of a branded GLP-1RA when advertising or referring to the compounded GLP-1RA? (does not include using an image of the branded GLP-1RA when selling the branded GLP-1RA)	yes, no
Does the website use/refer to the branded GLP-1RA in another way in relation to the compounded GLP-1RA? (does not apply to selling the branded GLP-1RA)	Open answer
Does the website refer to the compounded GLP-1RA as "generic"? (does NOT include generic liraglutide from Teva Pharmaceuticals)	yes, no
Does the website state anywhere that the compounded GLP-1RA is compounded?	yes, no
When referring to the compounded GLP-1RA, does the website always state that it is compounded within the same sentence or phrase? (instead of, for example, just saying semaglutide)	yes, no
Does the website provide a definition for compounding?	yes, no
Does the website state that the compounded GLP-1RA is NOT approved by the FDA?	yes, no
Does the website provide information indicating that FDA-approved products	yes, no

© 2025 Chetty AK et al. *JAMA Health Forum*.

containing semaglutide/ tirzepatide/ liraglutide are available?	
Does the website state or imply that the compounded GLP-1RA is FDA approved? (ex. saying "semaglutide is FDA approved" without further explanation)	yes, no
Does the website/seller state that it is "certified" or make a similar claim of certification?	yes, no
If yes to above, who certified the website/seller?	Open answer
Does the website state that the compounding facility it works with is "accredited" or make a similar claim of accreditation?	yes, no
If yes to above, who accredited the compounding facility?	Open answer
Which of the below indications, if any, does the website claim the compounded GLP-1RAs can be used for?	Glucose management in T2D (liraglutide, semaglutide, or tirzepatide); weight management in overweight/obesity (liraglutide, semaglutide, or tirzepatide); cardiovascular risk reduction in T2D (liraglutide or semaglutide); cardiovascular risk reduction in overweight/obesity (semaglutide); NA; other [multiple selections allowed]
Is there a claim supporting use for indication(s) not on the FDA-approved label?	yes, no
If there is a claim for indication(s) not on the FDA-approved label, what is it?	Open answer
Does the website cite clinical trial evidence to support efficacy claims for the compounded GLP-1RA?	yes, no
Does the website cite clinical trial evidence to support safety claims for the compounded GLP-1RA?	yes, no
Does the website indicate that a healthcare provider can provide more information or provide an opportunity to ask questions to a provider?	yes, no
Does the website include/link to the FDA-approved drug label?	yes, no
Does the website mention any of the warning and precautions on the FDA-approved label?	yes, no

© 2025 Chetty AK et al. *JAMA Health Forum.*

Does the website mention any of the adverse events on the FDA-approved label?	yes, no
Does the website mention any of the contraindications and limitations of use on the FDA-approved label?	yes, no
Does the website provide any information on the effects of GLP-1RA discontinuation?	yes, no
Does the website make any statement about using diet and exercise with an GLP-1RA?	yes, no
Does the website make any comparative claim(s) about other interventions?	yes, no
If so, are all comparative claims included in the FDA-approved label?	yes, no, NA
Does the website require clinician involvement before dispensing the compounded GLP-1RA?	yes, no, unclear, other
If so, what type of clinician involvement is required?	completing a questionnaire that is later reviewed by a clinician; messaging; telehealth visit (call or video); virtual contact required, method unspecified; inperson visit; unspecified/unclear type of involvement; labwork; none of the above; NA; other [multiple selections allowed]

GLP-1RA: glucagon-like peptide-1 receptor agonist, or gastric inhibitory peptide/glucagon-like peptide-1 receptor agonist; FDA: Food & Drug Administration

© 2025 Chetty AK et al. *JAMA Health Forum*.

eReferences

1. *Federal Food, Drug, and Cosmetic Act, 21 U.S.C. § 353b(d)(1).*; 2013.
2. Medications Containing Semaglutide Marketed for Type 2 Diabetes or Weight Loss. Food and Drug Administration. 2024. Accessed September 12, 2024. https://www.fda.gov/drugs/postmarket-drug-safety-information-patients-and-providers/medications-containing-semaglutide-marketed-type-2-diabetes-or-weight-loss

© 2025 Chetty AK et al. *JAMA Health Forum*.
