# Supplementary material for: Online Advertising of Compounded Glucagon-Like Peptide-1 Receptor Agonists
Source: JAMA Health Forum. 2025 Jan 17;6(1):e245018. doi: 10.1001/jamahealthforum.2024.5018 (PMC11742527; doi:10.1001/jamahealthforum.2024.5018)
Supplement: Supplement 2. — Data Sharing Statement [file jamahealthforum-e245018-s002.pdf]

Data Sharing Statement

Chetty. Online Advertising of Compounded Glucagon-Like Peptide-1 Receptor Agonists. *JAMA Health Forum*. Published January 17, 2025. doi:10.1001/jamahealthforum.2024.5018

Data
Data available: Yes
Data types: Data (not involving human participants)
How to access data: Relevant data are available on reasonable request from the corresponding author.
When available: With publication

Supporting Documents
Document types: None

Additional Information
Who can access the data: Anyone requesting the data
Types of analyses: For any purpose
Mechanisms of data availability: With investigator support
